# Clonotypic Analysis of Immunoglobulin Heavy Chain Sequences in Patients with Waldenström's Macroglobulinemia: Correlation with *MYD88* L265P Somatic Mutation Status, Clinical Features, and Outcome

**DOI:** 10.1155/2014/809103

**Published:** 2014-08-14

**Authors:** Loizos Petrikkos, Marie-Christine Kyrtsonis, Maria Roumelioti, George Georgiou, Anna Efthymiou, Tatiana Tzenou, Panayiotis Panayiotidis

**Affiliations:** Hematology Section of First Department of Propaedeutic Medicine, National and Kapodistrian University of Athens Medical School, Laikon Hospital, Agiou Thoma 17, 11527 Athens, Greece

## Abstract

We performed *IGH* clonotypic sequence analysis in WM in order to determine whether a preferential *IGH* gene rearrangement was observed and to assess *IGHV* mutational status in blood and/or bone marrow samples from 36 WM patients. In addition we investigated the presence of *MYD88* L265P somatic mutation. After *IGH* VDJ locus amplification, monoclonal VDJ rearranged fragments were sequenced and analyzed. *MYD88* L265P mutation was detected by AS-PCR. The most frequent family usage was *IGHV3* (74%); *IGHV3-23* and *IGHV3-74* segments were used in 26% and 17%, respectively. Somatic hypermutation was seen in 91% of cases. *MYD88* L265P mutation was found in 65,5% of patients and absent in the 3 unmutated. These findings did not correlate with clinical findings and outcome. Conclusion. *IGH genes'* repertoire differed in WM from those observed in other B-cell disorders with a recurrent *IGHV3-23* and *IGHV3-74* usage; monoclonal *IGHV* was mutated in most cases, and a high but not omnipresent prevalence of *MYD88* L265P mutation was observed. In addition, the identification of 3 patients with unmutated *IGHV* gene segments, negative for the *MYD88* L265P mutation, could support the hypothesis that an extra-germinal B-cell may represent the originating malignant cell in this minority of WM patients.

## 1. Introduction

Waldenström macroglobulinemia (WM) is an uncommon B-cell lymphoproliferative disorder characterized by bone marrow lymphoplasmacytic infiltration and by the presence of a monoclonal IgM immunoglobulin in the serum [1]. It belongs to the lymphoplasmacytic lymphoma type [2]. Clinical manifestations of WM include lymphoma-related lymphadenopathy, organomegaly, fatigue, disease related fever, or symptoms related to bone marrow failure (cytopenias) and IgM-related cryoglobulinemia, cold agglutinin syndrome, demyelinating neuropathy, and symptomatic hyperviscosity [3]. The monoclonal IgM is produced by malignant B-cells harboring a unique clonotypic rearrangement of immunoglobulin heavy chain variable genes (*IGH*), the VDJH rearrangement, associated with a specific constant region [4, 5]. On immunophenotype WM lymphoplasmacytes are usually CD5−, CD10−, CD23−, CD19+, CD20+ but numerous variations can be observed and in addition there is no characteristic pathognomonic genetic alteration; thus, differential diagnosis with other entities that can secrete IgM, share the same immunophenotype, and present lymphoplasmacytic differentiation may be sometimes difficult [6].

Immunoglobulin heavy chain gene (*IGH*) sequence analysis can provide useful clues in the investigation of B-cell lymphoproliferative disorders. It provides evidence regarding the maturation status of specific B-cell entities. Disorders characterized by germline immunoglobulin genes are likely to be derived from naive B cells, which have not encountered antigen. Most of B-cell lymphoproliferative disorders, however, exhibit somatic hypermutation (SHM) of immunoglobulin variable genes and are, therefore, derived from cells that have encountered antigen in germinal center. In addition, in chronic lymphocytic leukemia (CLL), marginal zone lymphoma (MZL), and mantle cell lymphoma (MCL), biased usage of* IGHV* genes and stereotyped clusters of immunoglobulin receptor support the role of antigen-driven mechanisms in their pathogenesis [7, 8]. The WM* IGHV* gene repertoire is completely different from other B-cell lymphoproliferative disorders like CLL and MZLs, as it is characterized by an overrepresentation of* IGHV3-23* genes with high* IGHV* mutation rates [8–13]. These features indicate that the transformation leading to WM occurred in postgerminal center B-cells that bear SHM and have been submitted to T-dependant antigen selection.

Recently, whole genome sequencing in WM patients revealed a highly recurrent somatic mutation (*MYD88* L265P) in these patients [8, 14–20]. It was furthermore suggested that* MYD88* L265P mutation possibly constitutes the initiating event, responsible for disease transformation [21, 22]. Furthermore its detection could constitute a valuable differential diagnosis tool.

In the present study we characterized* IGH* genes rearrangements and somatic hypermutations (SHM) in a cohort of WM patients and we investigated any eventual correlation with patients' clinical features. The frequency of the* MYD88* L265P mutation was also investigated and correlated with the* IGH* genes rearrangements in an attempt to reveal new insights in WM pathogenesis and the nature of WM B-cell.

## 2. Materials and Methods

### 2.1. Patients

A cohort of 36 WM patients was studied retrospectively. Diagnostic workout included physical examination, hematological and laboratory parameters, chest radiographs, and computed tomography scans of the thorax, abdomen, and pelvis. Bone marrow smears and biopsy as well as immunophenotype were performed in all patients, and lymph node histology was additionally performed in the cases with lymphadenopathy. International diagnostic criteria were used for the diagnosis of WM. Patients' characteristics are shown in Table 1.

Forty-five percent, 39% and 16%, of patients were staged 1, 2, and 3, respectively, according to IPSS [23]. Seventy-two percent were symptomatic and required treatment; median time to treatment and overall survival were 13 and 61 months, respectively.

The study was approved by the local ethical committee.

### 2.2. Specimens and DNA Extraction

We analyzed genomic DNA extracted from patients' blood and/or bone marrow samples (bone marrow mononuclear cells, bone marrow smears, and bone marrow biopsies).

Genomic DNA was extracted by standard protocols using QIAmp DNA Mini kit (QIAGEN) according to the manufacturer's recommendations.

### 2.3. Sequencing of IGHV Gene Sequences and Analysis of IGHV Sequences

Immunoglobulin heavy chain VDJ locus was amplified by PCR using the Biomed-2 strategy with FR1 primers as previously described [24]. It was possible to confirm monoclonality of the PCR product in 26/36 samples studied, by using capillary electrophoresis in Agilent 2100 Bioanalyzer using Agilent DNA 1000 kit (Agilent Technologies) according to the manufacturer's recommendations. PCR products were directly sequenced on both strands with the same primers using Sanger's chain-termination method and fluorescent dideoxynucleotides with GenomeLab DTCS Quick start kit in Beckman-Coulter CEQ 8000 sequencer platform.

In order to confirm monoclonality in the remaining 10/36 samples, cloning techniques were used as follows: (1) ligation of the PCR product to pCRII-TOPO vector (Invitrogen) using TOPO TA Cloning Kit (Invitrogen) according to manufacturer's recommendations, (2) transformation of One Shot TOP10 Chemically Competent* E. coli* cells (Invitrogen) by insertion of plasmids, (3) selection of 8–10 colonies of transformed* E. coli* cells followed by liquid culture (in LB medium for 12–14 hours at 37°C), and (4) plasmid DNA purification using PureLink HiPure Plasmid DNA Purification Kit (Invitrogen) according to manufacturer's recommendations. Plasmid DNA was sequenced, as described above, and sequences (8–10) were aligned using DNASTAR SeqMan Pro software in order to confirm monoclonality by detecting the same* IGH*-VDJ rearrangement in at least 3 out of 8 sequences.

Each clonal DNA* IGHV* sequence was aligned with the closest germline sequence using the international immunogenetics information system (IMGT, http://www.imgt.org/). Sequences were translated into amino acids in order to identify the functional* IGHV* gene rearrangement. The percentage of homology between the functional* IGHV* segment used in the tumor and the germline sequence was then calculated (excluding primer sequences). Somatic hypermutation was defined as a >2% deviation from germline (as per convention) [25]. The length of CDR3 regions was determined according to IMGT numbering.

### 2.4. Screening for* MYD88* L265P Mutations

Samples from 31 patients were also investigated for detection of* MYD88* L265P mutation by allele specific PCR (AS-PCR). Two PCRs were performed for each sample, one for wild type* MYD88* and one for possible detection of mutated* MYD88* by using primers as previously described [18]. PCRs were carried out by using a HotStarTaq DNA Plus Master Mix kit (QIAGEN). PCR consisted of an initial denaturation step of 15 minutes at 95°C, followed by 35 cycles of 95°C for 30 seconds, 58°C for 30 seconds, and 72°C for 30 seconds, with a final extension step of 5 minutes at 72°C. Agarose gel (1,5%) electrophoresis was performed to visualize the PCR products (220 bp).

### 2.5. Statistical Analysis

The SPSS software v.15 was used. Correlations between molecular findings and clinical parameters were assessed by the Mann-Whitney or by the chi-square test. Pictorial representation of survival curves was done by the Kaplan-Mayer method and their comparison by the log-rank test.

## 3. Results and Discussion

### 3.1. IGHV Usage and Mutation Analysis

**Figure 1:**
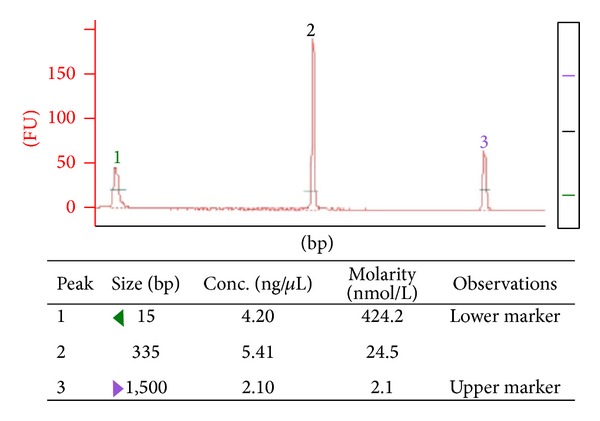
Electropherogram—after capillary electrophoresis in Agilent 2100 Bioanalyzer using Agilent DNA 1000 kit (Agilent Technologies)—of* IGHV6*-PCR product in one of the two patients of whom genomic DNA was extracted from blood sample and not bone marrow. Monoclonality of* IGHV*-PCR product is obvious (peak number 2) and was further confirmed by direct sequencing.

Thirty-six WM patients were studied. Two out of the 36 patients were excluded from the analysis, as in these two patients genomic DNA was extracted from peripheral blood and not bone marrow. Although these two patients had not lymphoma cells in blood (by morphology or immunophenotype analysis), monoclonality of* IGHV*-PCR product was confirmed in both samples (Figure 1 illustrates the electropherogram of* IGHV6*-PCR product in one of these two patients after capillary electrophoresis). Mutated* IGHV* genes were detected in these two patients (one* IGHV3-74* and one* IGHV6-1*).

We observed an* IGHV3* overrepresentation (74,3%), as high as described in previous studies [8, 12] but lower than reported by others [11, 26]. The distinctive* IGHV* gene segments usage in our patients is presented in Table 2. It should be mentioned that in one patient two different productive* IGH* VDJ sequences were identified (two different B-cell clones). There was an overrrepresentation of* IGHV3-23* gene usage (25,7%), as expected according to previous studies; while* IGHV3-74* gene -another member of the* IGHV3* family- was also overrepresented (17,1%), which is not reported in other studies [12, 26]. The repertoire of* IGHV* genes in WM, as presented in this study and previous studies, has some similarities with IgM-MGUS* IGHV* genes' repertoire [12, 13], but it is absolutely different from the ones observed in CLL/SLL [7], MCL [27], and MZL [8, 28, 29]. This is important because the aforementioned B-cell malignancies can secrete a monoclonal IgM and be in some cases misdiagnosed as WM or vice versa.

SHM was seen in all but three cases (91,4%). Mean percentages of mutations in all cases,* IGHV3* family,* IGHV3-23*, and* IGHV3-74* segments were 7,5%, 8%, 9,4%, and 7,5%, respectively (Table 3). These findings are in agreement with previous studies [11, 12, 26] and suggest that WM cells are derived from postgerminal center memory B cells that have been submitted to T-dependant antigen selection. It should be mentioned that in this study unmutated* IGHV* genes (≤2% deviation from germline homolog gene) were detected in 3 cases, as it was described in previous studies [10–12, 26], while in some other studies [8, 9, 13] all (100%) were mutated. In detail, 3 (8,6%) unmutated IGHV genes were detected (two* IGHV3-33* and one* IGHV5-51*), and one of the three was 100% homolog to germline gene. It is remarkable that none of these three genes belonged to the highly represented* IGHV* segments in WM (*IGHV3-23* and* IGHV3-74*). The existence of unmutated* IGHV* genes could mean that the transformation leading to WM does not target exclusively postgerminal center B-cells that bear SHM and have been submitted to T-dependent antigen selection. Even higher percentages of unmutated* IGHV* genes have been observed in resembling diseases such as splenic MZL (SMZL) [30–32]. Indeed an erroneous diagnosis can never be excluded in this disease although the 3 patients presented typical WM features. CDR3 length was short (≤17 amino acids) in 80% of all cases as previously described [8, 10–12, 33], although in the three unmutated cases the mean of CDR3 length was 22,3 amino acids.

The above-mentioned findings were compared with patients' physical and routine laboratory workup results and no correlations were found nor was it the case with time to treatment or survival. Although in CLL the* IGHV* genes mutational status is one of the most important independent prognostic factors [34–37], this was not the case for our WM patients.

### 3.2. *MYD88* Mutation Analysis

Since* MYD88* has been reported to be mutated (L265P) in the large majority of WM patients, we next looked for this mutation. Two out of the 31 patients investigated for detection of* MYD88* L265P mutation were excluded from the analysis, as in these two patients genomic DNA was extracted from blood and not bone marrow. However, in these samples monoclonality of* IGHV*-PCR product was confirmed. These two samples were negative for* MYD88* L265P mutation. Nineteen out of 29 patients (65,5%) were positive for the* MYD88* L265P mutation. This percentage is high and in accordance with the study of Gachard et al. [8]; however it is lower compared to other studies reporting a* MYD88* L265P mutation prevalence of 79% to 100% [14–16, 18, 20]. These variations could reflect differences in methodology followed by each study. The use, in our study, of BM unselected tissue for* MYD88* L265P AS-PCR assay could have contributed to the seemingly lower detection rate as has been raised by others. Cases not exhibiting* MYD88* L265P mutation had a statistically significant lower bone marrow infiltration by lymphoplasmacytes (*P* < 0.005).

The findings were also compared with patients' physical and routine laboratory workup results and no correlations were found nor was it the case with time to treatment or survival, as also described by Jiménez et al. [16]. We have also seen a difference in* MYD88* L265P detection based on bone marrow involvement, with a higher BM involvement in* MYD88* L265P positive patients.

Finally it should be mentioned that the mean of SHM levels of* IGHV* genes in* MYD88* L265P positive patients was 8,3%, while in* MYD88* L265P negative patients it was 5,4%. Five of seven patients with* IGHV3-23* who were tested for* MYD88* L265P and five out of five patient with* IGHV3-74* were positive for this mutation, which suggests that* IGHV3-23* and* IGHV3-74* are represented more in* MYD88* L265P positive patients. This may reinforce the concept that there are biological differences between the patients with and without the* MYD88* L265P mutation. This is further supported by an additional observation in this study: all three cases of unmutated* IGHV* genes were negative for* MYD88* L265P. These findings imply that a different (extra-germinal) B-cell represents the origin of the malignant cell in a minority of patients. Such hypothesis of the origin of the malignant cell in some WM patients is also described by Sahota et al. [38]. Larger studies may support further this concept.

In addition, the landscape is still unclear in this field as* MYD88* L265P mutation was recently found in other lymphoma entities [16, 18] while it was negative in lymphoplasmacytic lymphoma not secreting IgM [39]. Further studies are needed.

## 4. Conclusions

WM* IGH* genes repertoire, as expected, differs from that observed in normal B-cells and other B-cell diseases such as MZL, MCL, and B-CLL/SLL.

In addition to the known hyperrepresentation of the* IGHV3-23* gene, another member of the* IGHV3* family, the* IGHV3-74* gene is also overrepresented in WM, as shown in the present study. The high* IGHV* mutation rate supports a derivation of WM cells from postgerminal center memory B cells in the majority (91,4%) of WM patients. However, the identification of a minority of patients (3 of 34) with unmutated* IGHV* gene segments, negative for the* MYD88* L265P mutation, supports the hypothesis that they represent a subgroup of WM not arising from postgerminal B cells with a different disease pathogenesis. Finally, consensus and guidelines for* MYD88* L265P detection's methodology are needed, as it is quite obvious that this mutation could be both helpful in the diagnosis of WM and a potential therapeutic target in WM patients.

## Figures and Tables

**Table 1:** Clinical and laboratory findings for the study's WM patients.

	Mean value (median value)	Range
Age (years)	65,5 (64)	42–84
Gender (male/female)		
IPSS stage	19/17	
1	45%	
2	39%	
3	16%	
Bone marrow involvement	46,7% (40%)	5–100%
Lymphadenopathy	21%	
Splenomegaly	19%	
Hepatomegaly	9%	
Extranodal sites	3%	
IgM (mg/dL)	2777,2 (2500)	138–7870
Hb (g/dL)	10,9 (11,1)	6–14,3
Platelets (×10^9^/L)	233,2 (234)	60–472
WBC (×10^9^/L)	7,1 (6,7)	2,1–16,8
B_2_M (mg/dL)	4,1 (3,4)	1,9–10,4
Abnormal (high) LDH	27%	

**Table 2:** Distinctive *IGHV* gene segments usage in present study.

Segment	Number of patients	%
*IGHV1-2 *	1	2,86
*IGHV1-8 *	1	2,86
*IGHV2-5 *	1	2,86
*IGHV3-7 *	3	8,57
*IGHV3-23 *	9	25,71
*IGHV3-30 *	3	8,57
*IGHV3-33 *	2	5,71
*IGHV3-48 *	1	2,86
*IGHV3-64 *	1	2,86
*IGHV3-73 *	1	2,86
*IGHV3-74 *	6	17,14
*IGHV4-34 *	3	8,57
*IGHV4-59 *	2	5,71
*IGHV5-51 *	1	2,86

**Table 3:** Mean (median) of somatic mutations' percentage in different groups.

	Mean (median) somatic mutations' percentage	Range (%)
In all 35∗ cases	7,5% (7,3%)	0–16,1
In 32 cases with mutated genes (<98% homology)	8,1% (7,6%)	2,83–16,1
In *IGHV3* cases' group (27 cases)	8% (8,3%)	0–14,46
In *IGHV3-23* cases' group (9 cases)	9,4% (9,7%)	2,83–14,46
In *IGHV3-74* cases' group (6 cases)	7,5% (8,1%)	4,02–9,65

∗34 patients, 1 with two clones; *IGHV3-74* and *IGHV4-59*.
